# Quantification of Morpholine in Peel and Pulp of Apples and Oranges by Gas Chromatography−Mass Spectrometry

**DOI:** 10.3390/foods9060746

**Published:** 2020-06-05

**Authors:** Kunho An, Inhwan Kim, Chan Lee, Joon-Kwan Moon, Hee-Jae Suh, Jihyun Lee

**Affiliations:** 1 Department of Food Science and Technology, Chung-Ang University, Anseong 17546, Korea; kunho207@naver.com (K.A.); hgodos@daum.net (I.K.); chanlee@cau.ac.kr (C.L.); 2 Department of Plant Life and Environmental Science, Hankyong National University, Anseong 17579, Korea; jkmoon@hknu.ac.kr; 3 Department of Food Science, Sun Moon University, Asan-si, Chungcheongnam-do 31460, Korea; suhhj@sunmoon.ac.kr

**Keywords:** morpholine, peel, food additives, apples, citrus, oranges, GC-MS

## Abstract

Morpholine salts of fatty acids have been used in wax coatings on the surfaces of fruit and vegetable commodities in China and the United States, etc. However, morpholine usage was prohibited in many other countries because of safety concerns. We optimized analytical methods to determine morpholine in the peel and pulp of fruits and vegetables by gas chromatography-mass spectrometry (GC-MS). This morpholine analysis method was applied to real samples of apples, citrus fruits, and vegetables from Korea, China, and the U.S. The method was validated using apple and citrus fruit peels and pulp. The method detection limit (MDL) was 1.3–3.3 µg/kg. The recovery rates of morpholine were 88.6–107.2% over a fortified level of 10–400 µg/kg. Intra-day and inter-day precisions were 1.4–9.4% and 1.5–2.8%, respectively. The morpholine concentrations were n.d. (not detected)–11.19 and n.d. (not detected)–12.82 µg/kg in apple and citrus peels, respectively. Morpholine was not detected in citrus or apple pulp samples or in vegetable samples.

## 1. Introduction

Wax coating is applied to fruits and vegetables before shipping them over long distances. This reduces the quality loss of fruits and vegetables between harvest and consumption by retaining moisture and preventing weight loss. The coating is also applied to fruits at set periods after flowering to prevent damage to fruit during ripening. Fruit wax coating protects against sun damage in the summer by preserving the color of fruit. However, the wax coating is difficult to remove by typical washing of fruits and vegetables; thus, wax coatings can be absorbed in the body without degradation [1]. Active compounds such as ascorbic acid, malic acid, calcium chloride, calcium lactate, citric acid, and glutathione are often incorporated in the formulations of edible wax coating matrix (e.g., apple puree/pectin alginate, whey protein concentrates, alginate/apple puree, whey protein concentrates, and beeswax, etc.) of apple fruits to improve shelf life [2]. Morpholine is a colorless secondary amine ether that has been used in the edible coating matrix [3]. Morpholine has been used as a salt of fatty acid form (e.g., stearate salt) as an emulsifier to wax coating [4,5,6]. Morpholine has been added to the wax coating applied thinly and evenly to the fruit and vegetable surface [7]. However, there are safety concerns regarding the use of morpholine. Animals exposed to morpholine showed liver and kidney damage [8]. During digestion in the gut, morpholine can undergo nitrosation with excess nitrites from the diet to form *N*-nitroso-morpholine (NMOR), a genotoxic carcinogen in rodents [9]. A previous study showed that when morpholine was added to human saliva, NMOR was formed [10]. NMOR may be formed in the human gut when morpholine-treated fruits and vegetables are consumed, especially when they are consumed with wax-coated peel. The safety of morpholine has been extensively examined in many countries. Therefore, it has not been approved for use in the European Union [11]. Health Canada set morpholine acceptable daily intake as 0.48 mg kg^−1^ of body weight (bw) day^−1^ based on no observed adverse effect level as 96 mg kg^−1^ of bw day^−1^ in a chronic oral toxicity study [9]. Morpholine is approved for use in the United States and was included on the Environmental Protection Agency (EPA) master list and as an EPA registered pesticide in 1996 [8]. Canada and Australia have permitted the use of morpholine as fruit coating additive [9]. Furthermore, morpholine use for coating fruit was permitted in Chile and South Africa [3].

Recently, morpholine-free waxes have been introduced to avoid health concerns; however, the residues are present in package lines because morpholine is also used for various purposes such as fungicidal coating of paper. In China, Korea, and Japan, morpholine salts of fatty acids have been approved for coating the surface of fruit and vegetable commodities at Good Manufacturing Practice maximum levels [12,13,14].

In Korea, apple production increased from 474,712 tons to 545,349 tons from 2014 to 2017 [15]. Citrus production was 597,294 tons in 2017 [15]. Morpholine has been used as a coating component of apple and citrus [16]. Therefore, risk assessment of morpholine is necessary. The development of a method of analyzing for morpholine in apple and citrus matrix should also be essential before conducting a risk assessment of morpholine.

Methods for analyzing morpholine fatty acid salts are complex because it is linked to fatty acids such as stearate. The official standard analysis method of morpholine salts in fatty acids in Japan detects fatty acids rather than morpholine [13]. However, fatty acids are also present in fruit peel, so even if fatty acids are detected, they may be from the intrinsic fruit peel, not from morpholine fatty acid salts. Recent studies have used morpholine as a target analyte [17,18]. To analyze morpholine in fruit and vegetable matrix, various sample preparation methods were used. For example, the cleanup method (i.e., dispersive micro solid phase extraction) was included in a previous study [17,19,20,21]. In the previous study, dispersive micro solid phase extraction was conducted using PCX (polymer cation exchange) powder that can adsorb alkaline chemical compounds [17]. Furthermore, a dual solid phase extraction cartridge system was utilized for morpholine analysis in pineapple [22]. However, the system was time-consuming and costly [22]. In another previous study, no cleanup method was included [18].

In previous studies, gas chromatography coupled with a thermal conductivity detector and liquid chromatography coupled with a thermal energy analyzer were used to analyze morpholine in various matrices [23,24]. The thermal energy analyzer detects nitrosamines on the basis of chemiluminescence produced by the decay of the NO_2_ group when it is electronically excited [25,26]. However, the thermal conductivity detector and thermal energy analyzer have higher limits of detection than other detectors such as the flame ionization detector or mass spectrometry [27]. In a recent study of morpholine analysis by ultrahigh-performance liquid chromatography–high-resolution mass spectrometry (UHPLC-HRMS), the limit of detection of morpholine was reported as 2 µg/kg [17]. The method used whole fruits rather than separately analyzing the peel and pulp of fruit samples [17,18]. A previous study developed a gas chromatography-mass spectrometry (GC-MS) method for morpholine analysis in apple juice and whole apples [28] but was not used to examine the fruit peel itself. Because of the high lipid contents in fruit peel, lipids may lower extraction efficiency. Particularly, citrus fruit peels have a lipid content of 4.4% [29]. Moreover, previously reported methods for analyzing morpholine often did not use internal standards [18]; food samples often have a matrix effect, and thus more accurate analysis can be achieved when internal standards are used, particularly isotope-labeled standards which have very similar chemical structures to the target analyte.

There is little information on morpholine content in fruit commodities. Recently, Chen et al. reported morpholine residues of 80.5–598.7 μg/kg for citrus and 43.4–328.2 μg/kg for apples, respectively [17]. However, in this method, the peel was not separated from the pulp. Thus, the origin of the morpholine residues was unclear.

To address this issue, we developed a method for analyzing morpholine in the peel and pulp of fruits using GC-MS for the first time. As an internal standard, d8-morpholine was used. Additionally, the method was optimized by involving a lipid removal step and a different pH during the derivatization step. The validation results (i.e., method detection level (MDL), method quantification level (MQL), linearity, accuracy, precision, cross lab validation, measurement uncertainty, etc.) are presented. Additionally, morpholine monitoring was performed on 30 apples and citrus samples purchased from local markets in three countries, Korea, China, and the U.S., after separating the peel and pulp of the fruits. Vegetable samples (cucumber, squash, and paprika) were also analyzed.

## 2. Materials and Methods

### 2.1. Chemicals and Reagents

Morpholine standard (99.9% purity) and d8-morpholine (98.0% purity) were purchased from Sigma-Aldrich (St. Louis, MO, USA) and C/D/N Isotope, Inc. (Pointe-Claire, QC, Canada), respectively. Sodium nitrite, n-hexane, dichloromethane, HPLC-grade methanol, and fatty acid methyl ester standards were purchased from Sigma-Aldrich. Hydrochloric acid was purchased from Junsei Chemical Co. (Tokyo, Japan). Ultrapure water was prepared using a Milli-Q water system (Millipore, Billerica, MA, USA).

### 2.2. Sample Collection

Seventeen apple samples, 7 orange samples, 2 mandarin samples, 1 cucumber, 1 squash, and 1 paprika sample were purchased from local markets from Anseong in South Korea, Beijing in China, and Boston in the U.S. The oranges purchased from Korea were produced in the U.S.A. or Australia and imported to Korea. Other fruits and vegetables purchased from Korea were produced in Korea.

More than 50 fruits or vegetables per sample were purchased to prepare a composite sample. After purchase, the (unwashed) apple and citrus fruits were peeled and separated into peel and pulp. After peeling, each fruit was sliced into six pieces and two pieces were used for making a composite sample for pulp. For cucumber, squash, and paprika, whole vegetables were used for analysis. They were diced and one third of the diced pieces were used to make a composite vegetable sample. The composite samples were lyophilized and stored at −80 °C until analysis within 1 month. The freeze-dried samples were ground before the analysis.

### 2.3. Optimization of Sample Preparation Method for Morpholine Analysis in Fruit Peel and Pulp

A previously described sample preparation method for morpholine was modified by adding a lipid removal step and changing the pH during the derivatization step [28]. Sequential extraction was performed. The first step employed the lipid removal method and the second step involved a derivatization step to *N*-nitroso-morpholine. As described in the Introduction, Cao et al. prepared samples of apple juice rather than of fruits [28]. When the method of Cao et al. was used for fruit samples, particularly fruit peels, the final extract solution was unclear, possibly because of the presence of lipids. Therefore, after spiking morpholine standard, lipids were removed from the fruit samples as follows. First, 18 mL of nano-pure water was added to 2.0 g of freeze-dried fruit (apple peel, apple pulp, citrus peel, or citrus pulp) powder in a 15 mL tube. Then, morpholine and d8-morpholine were added. The final isotope labelled internal standard concentration was 100 μg/kg fruit dry weight (DW). The tube was vortexed for 15 min, ultrasonicated for 15 min, and centrifuged at 19,587× *g* for 10 min. Next, 17 mL of n-hexane was added to 5 mL of the supernatant, followed by vortex mixing for 15 min. After centrifugation at 9598× *g* for 10 min, the n-hexane layer was removed, and the non-organic layer was collected. The lipid removal step was repeated twice more using the sample residue.

Morpholine was spiked and analyzed by GC-MS after defatting and derivatization of the fruit samples. By adding the defatting step to the previous method, the final extract solution was clear. For derivatization, 200 μL of 0.05 M HCl and 200 μL of saturated sodium nitrite were added to 2.0 mL of the defatted sample extract and the mixture was vortexed for 30 s. HCl was added to improve recovery and the pH was optimized by adding different amounts of HCl. Herein, different pH levels (pH 1.5, 3.0, and 6.5) were tested and recovery was compared. To optimize the derivatization method, accuracy and precision were determined at pH 1.5, 3.0, and 6.5. Recovery of pH 6.5 was 124.3%, whereas, the recoveries of morpholine stearate at pH 1.5 and 3.0 were 102.2% and 106.5%, respectively. The precision (relative standard deviation, RSD%) of morpholine stearate measurements at pH 1.5, 3.0, and 6.5 were 0.8, 11.6%, and 7.0%, respectively. The morpholine recovery% and RSD% were best at pH 1.5. Thus, pH 1.5 should be used to analyze morpholine.

Then, the extract was heated at 40 °C for 5 min and cooled. Finally, 0.5 mL of dichloromethane was added, and the mixture was vortexed for 1 min and left to stand for 10 min. An aliquot of the organic layer was collected and placed in an amber vial after filtration through a 0.22-μm filter. All samples were stored at −20 °C before analysis, which was performed in 1–2 days.

### 2.4. Method Validation (Method Detection Limit, Method Quantification Limit, Linearity, Accuracy, Precision, Cross-Lab Validation, and Measurement Uncertainty)

For method validation, apple and oranges were purchased from a local market in Anseong, Korea. Apple peel, apple pulp, orange peel, and orange pulp were lyophilized and stored at −80 °C until analysis. This method was validated for MDL, MQL, linearity, accuracy, precision, cross-lab validation, and measurement uncertainty. The MDL and MQL were based on International Council for Harmonisation of Technical Requirements for Pharmaceuticals for Human Use (ICH) guidelines and calculated as LOD (limit of detection) = 3.3σ/S and LOQ (limit of quantification) = 10σ/S, where σ is the standard deviation and S is the slope of the standard curve [30]. To evaluate intra-day accuracy and precision, known amounts of morpholine were added to the four matrices at final concentrations of 10–400 μg/kg and measurements were repeated 5 times. For inter-day accuracy and precision, known amounts of morpholine were added to the four matrices at final concentrations of 25, 100, and 400 μg/kg for 3 days. Accuracy and precision were validated in three different laboratories for cross-lab validation according to ICH guidelines [30].

Measurement uncertainty was also estimated for the morpholine analysis method in apple peel, apple pulp, orange peel, and orange pulp using the combined standard uncertainty based on the Guide to the Expression of Uncertainty in Measurement Guide by KRISS [31] and EURA CHEM Guide [32]. The intra-laboratory data of the reference material, calibration curves, repeatability, and sample preparation were used for estimation. To obtain measurement uncertainty (U), a coverage factor of ~ 95%, where k = 2, was used [33].

### 2.5. Morpholine Analysis by GC-MS

Morpholine analysis was performed on a GC-MS instrument (7890A GC-5975 MSD, Agilent Technologies, Santa Clara, CA, USA). The analysis method was modified from that described by Cao et al. by changing the analytical column and split ratio, etc. [28]. Separation was carried out on a DB-1701 column (30 m × 0.25 mm i.d., 0.25 µm film thickness, Agilent Technologies). A DB-wax column (30 m × 0.25 mm i.d., 0.25 µm film thickness, Agilent Technologies) was compared to optimize the method; the DB-1701 column resulted in better separation with a better response (data not shown). The injection volume was 1 μL and the sample was vaporized at 250 °C with a 1:7 split ratio. The GC oven temperature was operated as follows: 100 °C for 4 min, heating to 120 °C at 10 °C/min and held for 3 min, and then heating to 250 °C at 20 °C/min and held for 5 min. The electron energy was 70 eV. The flow rate was 2.0 mL/min of He (99.999%). The transfer line temperature, quadrupole temperature, and electron impact ionization source temperature were held at 280 °C, 150 °C, and 230 °C, respectively. The scan rate was 3.2 scans/s. Four different ions were selected to detect and quantify *N*-nitroso-morpholine (qualifier ion: m/z 86; quantifier ion: m/z 116) and its isotope (qualifier ion: m/z 64; quantifier ion: m/z 124) in selected ion monitoring (SIM) mode.

## 3. Results and Discussion

### 3.1. Method Validation for Morpholine Analysis

In morpholine salts of fatty acids, fatty acid salts are attached to morpholine. Therefore, morpholine salts of fatty acids can be analyzed by (1) removing morpholine and analyzing only fatty acid or (2) removing fatty acid and analyzing only morpholine. Herein, we adopted the second method and morpholine analysis was performed by GC-MS.

**Figure 1:**
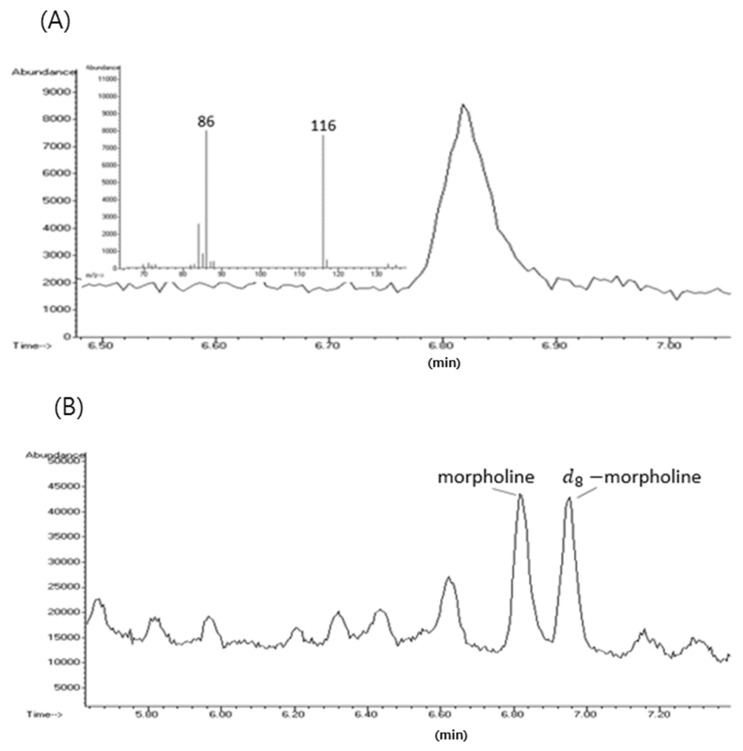
Representative selective ion chromatogram (SIM) of morpholine and mass spectrum of morpholine (**A**) and representative total ion chromatogram (TIC) of morpholine and d8-morpholine in apple peel sample (**B**).

Representative GC-MS selective ion monitoring (SIM) and total ion chromatogram (TIC) of morpholine in an apple peel sample are shown in Figure 1A,B. A mass spectrum is also shown in Figure 1. Because morpholine salts are often used in fruit wax coating applied to fruit skin, method validation for morpholine analysis in fruit peel is essential. This is the first study to validate the morpholine analysis method separately for the fruit peel and pulp.

Method validation was carried out by performing a spike recovery test of morpholine in the four matrices (apple peel, apple pulp, orange peel, and orange pulp). The results are shown in Table 1. The correlation coefficient (R^2^) of the calibration curves showed high linearity (>0.9999) over a linear range of 10–400 µg/kg. The MDL and MQL were calculated using statistical methods in the four matrices and showed values of 1.3–3.3 and 4.1–10.1 µg/kg, respectively. In previous studies using liquid chromatography-mass spectrometry (LC-MS), the MQL of morpholine in whole fruits of apples or citrus fruits were 5–10 μg/kg [17,18]. In a previous study using GC-MS, the MQL of morpholine in apple juice was 24.4 μg/L [28]. Thus, our method showed a similar MQL as published LC-MS methods and much lower MQL compared to a published GC-MS method [17,18,28].

Accuracy and precision of the morpholine analysis method (10–400 µg/g DW) were evaluated by intra-day and inter-day test and the result was expressed as the recovery rate% and relative standard deviation (RSD%) (Table 2). Intra-day recovery% (in 1 day, 5 replicates) and RSD% were 97.8–104.4% and 0.8–9.4%, respectively. The inter-day recovery% and RSD%, obtained by repetition for 3 days were 98.5–103.2% and 1.5–2.8%, respectively. Thus, accuracy and precision in this study were acceptable according to ICH guidelines. In a previous study using GC-MS, the morpholine recovery rate was 94.3–109.0% and intra-day and inter-day precision ranged from 2.3 to 4.4% and 4.8 to 5.2%, respectively, at fortified levels of 50–400 µg/L apple juice [28]. In a previous study using UHPLC-HRMS, the intra-day morpholine recovery% was 85.4–102.7% and 78.4–96.2% for apple and citrus matrices, respectively, at fortified morpholine concentrations of 5–100 µg/kg [17]. Thus, the method validation results for morpholine analysis revealed good linearity with wider linear range, low MDL and MQL, high accuracy, and high precision.

In this study, cross-validation studies for apple peel, apple pulp, orange peel, and orange pulp were conducted in three different laboratories of two universities and a food research institute according to International Council for Harmonisation of Technical Requirements for Pharmaceuticals for Human Use (ICH) guidelines (ICH, 2005), and RSD% ranged from 3.3% to 6.9% with recovery% ranging from 100.7% to 105.7% in the four matrices, satisfying the results of accuracy and precision of cross-laboratory cross-validation studies (Table 3).

The uncertainty of measurement of morpholine in the four matrices was estimated and the result is shown in Table 4. U1 (uncertainty associated with sample preparation) was calculated using a chemical balance and 25-mL measuring cylinder. Uncertainty in the chemical balance was measured with a certificate of calibration (0.0004 g), repeatability (0.0001 g), and stability (0.000037 g), and uncertainty in the measuring cylinder was measured with a certificate of calibration (1 mL), repeatability (0.14 mL), and variation in volume based on temperature (0.009 mL). The relative standard uncertainties of the chemical balance and 2-mL volumetric flask were 0.00005 and 0.00296, respectively. The combined uncertainty of sample preparation was 0.00651. U2 (uncertainty associated with the reference material) was measured according to a certificate of analysis with morpholine standard purity. The uncertainty of stock solution U3 was measured using a chemical balance and 0.2 and 1-mL pipette. The uncertainty of the 0.2-mL pipette was measured with a certificate of calibration (0.0006 mL), repeatability (0.00031 mL), and variation in volume based on temperature (0.0001 mL). The relative standard uncertainties of the 0.2 mL pipette and 1 mL pipette were 0.0008 and 0.0007, respectively. The combined uncertainty of the stock solution was 0.0065. The uncertainty of the calibration curve (U4) was obtained for the morpholine spike recovery test, which was measured at three concentrations in triplicate. The uncertainty of repeatability (U5) was evaluated to measure the average content of each morpholine in 20-g samples. Our results revealed measurement uncertainty values of 13.6%, 8.0%, 9.6%, and 7.5% for apple peel, apple pulp, orange peel, and orange pulp, respectively. The values are in an acceptable range under 20%.

### 3.2. Applying the Method to Real Samples (Fruits and Vegetables)

The developed method was used to determine the residual morpholine contents of peel and pulp of apple and citrus fruits purchased from domestic and foreign local markets. The averaged morpholine analysis data is shown in Table 5. Individual sample data is shown in Table 6. A total of 26 fruit samples were analyzed and detected in 11 fruit samples from: the peel of apples purchased from the USA, peel of oranges purchased from Korea, and peel of mandarins purchased in Korea. The morpholine levels in the positive samples, apple peel, orange peel, and mandarin peel were 1.15–11.19, 0.97–12.82, and 0.91–0.92 mg/kg, respectively. Morpholine was not detected in the peel of Korean apples, peel of China apples, or peel of China oranges. Because morpholine was not detected in the pulp of any fruits and detected only in the peel of several fruit samples, oranges and apples should be peeled before consumption to avoid exposure to morpholine residue in the peel. The residue may originate from packaging lines. Morpholine residue was detected only in the peel of apple and peel of citrus fruits at levels lower than those reported in the 1980s. The residue may originate from packaging lines. Chen et al. (2015). reported that 3 apples and 5 citrus samples from a total of 8 apples and 10 citrus samples were positive for morpholine residues, with contents of 0.43–3.3 mg/kg for apples and 0.81–5.99 mg/kg for citrus purchased from local markets in China after correcting fresh weight to dry weight based on a 90% estimated moisture content [17]. However, the values from the study of Chen et al. (2015) could not be compared to our results because they used whole fruits rather than separating the peel and pulp of fruits. In 1983, Ohnishi et al. reported that 22 samples of the peel of citrus fruits contained morpholine up to 71.1 mg/kg and their pulp contained up to 0.7 mg/kg [34]. Compared to this value, morpholine residues were decreased extensively because their concentrations in citrus peel decreased up to 5.99 mg/kg and no morpholine residue was found in the any fruit pulp samples. Additionally, no morpholine residue was detected in cucumber, squash, and paprika samples purchased from a local market from Anseong, Korea.

## 4. Conclusions

Herein, we developed a robust morpholine analysis method by involving a lipid removal step and a different pH during derivatization in peel and pulp of fruits. The method showed low MDL and high accuracy and precision with broad linear range. We also tested real samples purchased from local markets from Korea and foreign markets (U.S. and China). Morpholine residue was detected only in the peel of apple and peel of citrus fruits at levels lower than those reported in the 1980s. The residue may originate from packaging lines. This method can be used to monitor large numbers of samples.

## Figures and Tables

**Table 1:** Linearity, linear range, method detection level (MDL), and method quantification level (MQL) in four matrices (apple peel, apple pulp, orange peel, and orange pulp).

Matrix	Standard Curve ^a^	Linearity (R^2^)	Calibration Range (µg/kg)	MDL ^b^ (µg/kg)	MQL ^c^ (µg/kg)
Apple peel	y = 1.0280x − 0.0081	0.9999	10–400	3.3	10.1
Apple pulp	y = 1.0136x − 0.0041	1.0000	10–400	1.3	4.1
Orange peel	y = 1.0173x − 0.0035	1.0000	10–400	2.3	8.5
Orange pulp	y = 1.0126x − 0.0051	0.9999	10–400	2.9	8.7

^a^ X-axis is concentration ratio of morpholine/d8-morpholine and y-axis is response ratio of morpholine/d8-morpholine. ^b^ The MDL was calculated by 3.3 times standard deviation divided by the slope of the standard curve. ^c^ The MQL was calculated by 10 times standard deviation divided by the slope of the standard curve.

**Table 2:** Recovery and repeatability for morpholine in four fruit matrices.

***Intra-Day (n = 5)***								
**Morpholine Concentration (µg/kg)**	**Apple Peel**		**Apple Pulp**		**Orange Peel**		**Orange Pulp**	
	**Recovery (%)**	**RSD (%)**	**Recovery (%)**	**RSD (%)**	**Recovery (%)**	**RSD (%)**	**Recovery (%)**	**RSD (%)**
10	104.0	4.1	102.2	0.9	104.4	0.9	102.0	9.4
25	101.3	4.1	102.0	1.4	100.5	1.4	99.8	1.5
50	99.7	2.5	98.9	3.7	98.0	3.7	97.8	1.6
100	101.0	2.2	98.7	3.0	100.7	3.0	101.2	1.6
200	98.7	1.6	100.8	2.7	99.8	2.7	99.9	3.4
400	100.3	2.9	99.9	0.8	100.0	0.8	102.2	2.4
***Inter-Day (3 Days)***								
**Morpholine Concentration (µg/kg)**	**Apple Peel**		**Apple Pulp**		**Orange Peel**		**Orange Pulp**	
	**Recovery (%)**	**RSD (%)**	**Recovery (%)**	**RSD (%)**	**Recovery (%)**	**RSD (%)**	**Recovery (%)**	**RSD (%)**
25	101.3	2.8	99.8	1.9	98.5	1.9	103.2	1.9
100	99.8	2.2	99.7	2.0	100.5	2.0	100.0	1.5
400	99.4	2.2	100.4	1.8	99.6	1.8	99.1	2.3

RSD means relative standard deviation.

**Table 3:** Recovery and repeatability of morpholine analysis in four fruit matrices of cross-laboratory cross-validation studies.

Theoretical Morpholine Concentration (μg/kg DW)	Observed Concentration (μg/kg DW)			Recovery%	RSD%
	Lab A	Lab B	Lab C		
*Apple peel*					
25	25	27	25	102.7%	6.9
100	106	104	103	104.3%	4.0
400	416	433	418	104.1%	3.5
*Apple pulp*					
25	26	24	26	101.3%	5.7
100	101	112	104	105.7%	5.4
400	404	440	407	103.7%	5.3
*Orange peel*					
25	25	25	25	100.0%	3.4
100	96	107	102	101.7%	5.6
400	420	427	406	103.9%	3.5
*Orange pulp*					
25	26	27	25	104.0%	6.0
100	98	104	100	100.7%	6.2
400	421	420	414	103.4%	3.3

**Table 4:** Measurement uncertainty (U) obtained for the morpholine analysis in apple peel, apple pulp, orange peel, and orange pulp.

Matrix	U1 ^a^	U2 ^b^	U3 ^c^	U4 ^d^	U5 ^e^	U ^f^
Apple peel	0.0084	0.0116	0.0058	0.0374	0.0530	13.15
Apple pulp	0.0084	0.0116	0.0058	0.0301	0.0050	6.79
Orange peel	0.0084	0.0116	0.0058	0.0250	0.0270	6.67
Orange pulp	0.0084	0.0116	0.0058	0.0035	0.0110	3.27

The ^a^ individual uncertainties of the sample preparation (U1), ^b^ reference material (U2), ^c^ stock solution (U3), ^d^ calibration curve (U4), ^e^ repeatability (U5), and ^f^ expanded uncertainty (U).

**Table 5:** Averaged morpholine concentrations in fruit and vegetable samples.

	n	Morpholine Range (mg/kg DW)	Average Morpholine (mg/kg DW)		
*Apple peel*					
Korea	8	n.d. ^b^	n.d.		
USA	4	1.15–11.19	4.4	±	4.7
China	5	n.d.	n.d.		
*Apple pulp*					
Korea	8	n.d.	n.d.		
USA	2	n.d.	n.d.		
China	5	n.d.	n.d.		
*Orange peel*					
Korea	5	0.97–12.82	5.8	±	5.4
China	2	n.d.	n.d.		
*Orange pulp*					
Korea	5	n.d.	n.d.		
China	2	n.d.	n.d.		
*Mandarin peel*					
Korea	2	0.91–0.92	0.9	±	0.1
*Mandarin pulp*					
Korea	2	n.d.	n.d.		
*Cucumber* ^a^	1	n.d.	n.d.		
*Squash* ^a^	1	n.d.	n.d.		
*Paprika* ^a^	1	n.d.	n.d.		

^a^ Whole vegetable samples were used for morpholine analysis. ^b^ n.d. indicates not detected.

**Table 6:** Concentration of morpholine in fruit and vegetable samples.

Sample Information (Purchased Location)	Origin	Morpholine Content in Peel (mg/kg DW)			Morpholine Content in Pulp (mg/kg DW)
Apple1 (Korea)	Korea	n.d. ^a^			n.d.
Apple2 (Korea)	Korea	n.d.			n.d.
Apple3 (Korea)	Korea	n.d.			n.d.
Apple4 (Korea)	Korea	n.d.			n.d.
Apple5 (Korea)	Korea	n.d.			n.d.
Apple6 (Korea)	Korea	n.d.			n.d.
Apple7 (Korea)	Korea	n.d.			n.d.
Apple8 (Korea)	Korea	n.d.			n.d.
Apple9 (China)	Dalian, China	n.d.			n.d.
Apple10 (China)	Dalian, China	n.d.			n.d.
Apple11 (China)	Dalian, China	n.d.			n.d.
Apple12 (China)	Dalian, China	n.d.			n.d.
Apple13 (China)	Dalian, China	n.d.			n.d.
Apple14 (USA)	USA	3.97	±	0.10	n.d.
Apple15 (USA)	USA	11.19	±	0.37	n.d.
Apple16 (USA)	USA	1.25	±	0.25	n.d.
Apple17 (USA)	USA	1.15	±	0.02	n.d.
Orange1 (Korea)	California, USA	n.d.			n.d.
Orange2 (Korea)	Cobram, Australia	3.68	±	0.08	n.d.
Orange3 (Korea)	Cobram, Australia	12.82	±	0.79	n.d.
Orange4 (Korea)	Cobram, Australia	0.97	±	0.05	n.d.
Orange5 (Korea)	California, USA	n.d.			n.d.
Orange6 (China)	California, USA	n.d.			n.d.
Orange7 (China)	California, USA	n.d.			n.d.
Mandarine1 (Korea)	Jeju, Korea	0.92	±	0.09	n.d.
Mandarine2 (Korea)	Jeju, Korea	0.91	±	0.01	n.d.
Cucumber (Korea)	Korea	n.d.			n.d.
Squash (Korea)	Korea	n.d.			n.d.
Paprika (Korea)	Korea	n.d.			n.d.

^a^ n.d. indicates not detected.

## References

[1] Kuchowicz E., Rydzyński K. (1998). Risk assessment of morpholine (tetrahydro-2H-1, 4-oxazine): A time for reevaluation of current occupational exposure standards?. Appl. Occup. Environ. Hyg.

[2] Vakkalanka M., D’Souza T., Ray S., Yam K.L., Mir N., Yam K.L., Lee D.S. (2012). 7—Emerging packaging technologies for fresh produce. Emerging Food Packaging Technologies.

[3] Walker M.J., Gray K., Hopley C., Bell D., Colwell P., Maynard P., Burns D.T. (2012). Forensically robust detection of the presence of morpholine in apples—Proof of principle. Food Anal. Methods.

[4] McGuire R.G., Hagenmaier R.D. (1996). Shellac coatings for grapefruits that favor biological control of *Penicillium digitatum* by *Candida oleophila*. Biol. Control.

[5] El-Gamal I., Khidr T., Ghuiba F. (1998). Nitrogen-based copolymers as wax dispersants for paraffinic gas oils. Fuel.

[6] Njombolwana N.S., Erasmus A., Van Zyl J.G., du Plooy W., Cronje P.J., Fourie P.H. (2013). Effects of citrus wax coating and brush type on imazalil residue loading, green mould control and fruit quality retention of sweet oranges. Postharvest Biol. Tech..

[7] Kumar R., Kapur S. (2016). Morpholine: A glazing agent for fruits and vegetables coating/waxing. Int. J. Sci. Technol. Eng..

[8] USDA Morpholine Was Included in EPA (Environment Protection Agencies, United State). https://www.ams.usda.gov/sites/default/files/media/Morph%20Technical%20Advisory%20Panel%20Report.pdf.

[9] Health Canada, Archived—A Summary of Health Hazard Assessment of Morpholine in Wax Coatings of Apples. https://www.canada.ca/en/health-canada/services/food-nutrition/food-safety/information-product/summary-health-hazard-assessment-morpholine-coatings-apples.html.

[10] Tannenbaum S.R., Archer M.C., Wishnok J.S., Bishop W.W. (1978). Nitrosamine formation in human saliva. J. Natl. Cancer Inst..

[11] European Union Food Additives Database. https://eur-lex.europa.eu/legal-content/EN/TXT/?uri=CELEX:32008R1333.

[12] MFDS (Korea Ministry of Food and Drug Safety) Food Additives Code. https://www.mfds.go.kr/eng/brd/m_15/view.do?seq=72242&srchFr=&srchTo=&srchWord=code&srchTp=7&itm_seq_1=0&itm_seq_2=0&multi_itm_seq=0&company_cd=&company_nm=&page=1.

[13] MHLW (Ministry of Health, Labour and Welfare) Morpholine Salts of Fatty Acids. https://www.mhlw.go.jp/shingi/2005/10/dl/s1027-7g07b.pdf.

[14] MOH (Ministry of Health of the People’s Republic of China) Standards for Use of Food Additives. http://www.cirs-reach.com/China_Chemical_Regulation/GB_2760-2011_Food_Safety_National_Standards_for_the_Usage_of_Food_Additives.html.

[15] Korean Statistical Information Service. http://kosis.kr/index/index.do.

[16] Baldwin E.A. (1994). Edible coatings for fresh fruits and vegetables: Past, present, and future. Edible Coatings and Films to Improve Food Quality.

[17] Chen D., Miao H., Zou J., Cao P., Ma N., Zhao Y., Wu Y. (2015). Novel dispersive micro-solid-phase extraction combined with ultrahigh-performance liquid chromatography–high-resolution mass spectrometry to determine morpholine residues in citrus and apples. J. Agric. Food Chem..

[18] Hengel M.J., Jordan R., Maguire W. (2014). Development and validation of a standardized method for the determination of morpholine residues in fruit commodities by liquid chromatography–mass spectrometry. J. Agric. Food Chem..

[19] Xie W., Han C., Qian Y., Ding H., Chen X., Xi J. (2011). Determination of neonicotinoid pesticides residues in agricultural samples by solid-phase extraction combined with liquid chromatography–tandem mass spectrometry. J. Chromatogr. A.

[20] Kanrar B., Mandal S., Bhattacharyya A. (2010). Validation and uncertainty analysis of a multiresidue method for 42 pesticides in made tea, tea infusion and spent leaves using ethyl acetate extraction and liquid chromatography–tandem mass spectrometry. J. Chromatogr. A.

[21] Li Y., Wang M., Yan H., Fu S., Dai H. (2013). Simultaneous determination of multiresidual phenyl acetanilide pesticides in different food commodities by solid-phase cleanup and gas chromatography-mass spectrometry. J. Sep. Sci..

[22] Gros P., Matignon F., Plonevez S. (2011). Determination of morpholine in fruits using LC− MS/MS. Ann. Falsif. Expert. Chim. Toxicol..

[23] Sen N.P., Baddoo P.A. (1988). An investigation on the possible presence of morpholine and *N*-nitrosomorpholine in wax-coated apples. J. Food Saf..

[24] Hotchkiss J.H., Vecchio A.J. (1983). Analysis of direct contact paper and paperboard food packaging for *N*-nitrosomorpholine and morpholine. J. Food Sci..

[25] Fu C.G., Xu H.-D. (1995). High-performance liquid chromatography with post-column chemiluminescence detection for simultaneous determination of trace *N*-nitrosamines and corresponding secondary amines in groundwater. Analyst.

[26] Ikeda K., Migliorese K.G., Curtis H. (1990). Analysis of nitrosamines in cosmetics. J. Soc. Cosmet. Chem.

[27] Gao Y., Cao Y., Chen S.-L., Wang Y., Zhu R., Sun W., Zhang Q., Fan Y. (2017). Differences in product distribution measured with flame ionization detector gas chromatography and thermal conductivity detector gas chromatography during the dimethyl ether-to-olefins and methanol-to-olefins processes. Energ. Fuel.

[28] Cao M., Zhang P., Feng Y., Zhang H., Zhu H., Lian K., Kang W.J. (2018). Development of a method for rapid determination of morpholine in juices and drugs by gas chromatography-mass spectrometry. J. Anal. Methods Chem..

[29] Shan Y., Shan Y. (2016). Chapter 6—Drying of Citrus Peel and Processing of Foods and Feeds. Comprehensive Utilization of Citrus By-Products.

[30] ICH Validation of Analytical Procedures: Text and Methodology. https://www.ema.europa.eu/en/ich-q2-r1-validation-analytical-procedures-text-methodology..

[31] KRISS (Korea Research Institute of Standards and Science) ISO/IEC Guide 98-3:2008 Guide to the Expression of Uncertainty in Measurement. https://www.kriss.re.kr/w/fileDownload.do?fileSeq=1008.

[32] Eurachem Quantifying Uncertainty in Analytical Measurement. https://www.eurachem.org/images/stories/Guides/pdf/QUAM2012_P1.pdf.

[33] ISO2008 ISO/IEC Guide 98-3: 2008 (E) Uncertainty of Measurement-Part 3: Guide to the Expression of Uncertainty in Measurement. http://www.iec.ch/dyn/www/f?p=103%3A391%3A0%3A%3A%3A%3AP391_PUB_ID%2CP391_LANG%3A11961.

[34] Ohnishi T., Kubota M., Okada A., Tonami K. (1983). Residual survey investigation and removal efficiency by washing with kitchen detergent of food additive morpholine. Hokuriku Koshu Eisei Gakkaishi.

